# Prediction of spontaneous closure of ventricular septal defect and guidance for clinical follow‐up

**DOI:** 10.1002/clc.23173

**Published:** 2019-03-25

**Authors:** Xinyang Li, Weidong Ren, Guang Song, Xintong Zhang

**Affiliations:** ^1^ Department of Ultrasound Shengjing Hospital of China Medical University Shenyang China

**Keywords:** children, echocardiography, spontaneous closure, ventricular septal defect

## Abstract

**Aim:**

To predict the spontaneous closure of ventricular septal defect (VSD) and assist pediatrician to manage VSD children.

**Methods:**

Between January 2008 and December 2016, 132 children diagnosed with isolated VSD by echocardiography were enrolled. All participating children were followed‐up by echocardiography yearly until the closure of VSD or 6 years old. The clinical indicators and echocardiographic parameters of patients were collected. Statistically significant factors were used to establish a Logistic Regression model for predicting spontaneous closure of VSD. Receiver operating characteristic (ROC) analysis was used to assess the specificity and sensitivity of Logistic Regression model.

**Results:**

Spontaneous closure occurred in 60% of all patients; 57% in perimembranous VSD (p‐VSD) and 64% in muscular VSD (m‐VSD) patients. Initial diagnosis age, defect size, aneurysms tissue of the ventricular membranous septum (ATVMS), pulmonary hypertension (PH), and left ventricular diastolic dimension (LVDD) were statistically significant. Defect size, ATVMS and LVDD were determined by the Logistic Regression model as representative factor. P‐VSD and m‐VSD model had areas under the ROC curves 0.854 and 0.898, respectively.

**Conclusion:**

We inferred that defect size, ATVMS and LVDD were characteristic and representative predictors for spontaneous closure of VSD. And we summarized the prognostic factors and recommended a follow‐up criteria to assist the pediatrician managing VSD children.

## 1 INTRODUCTION

Ventricular septal defect (VSD) is the most common congenital heart disease (CHD), accounting for almost 50% of all CHD.^1^, ^2^ The estimated prevalence of VSD is 2 to 3.94 per 1000 live births diagnosed by echocardiography.^3^, ^4^ Two‐dimensional (2D) combined color Doppler echocardiography makes it easy to diagnose VSD, and to measure size and position of defect, as well as to evaluate the anatomical relationship with the surrounding tissue. The spontaneous closure rate of VSD remains controversial, however, VSD generally close spontaneously in 12% to 84% cases.^5^, ^6^, ^7^ This wide range may be attributed to age, size and position of VSD, diagnostic methods, and follow‐up period. Recent studies focus on describing the incidence and natural history of VSD.^6^ Others studies had a short follow‐up time.^8^ In this study, patients were followed‐up from the infant period to 6 years old, and showed the spontaneous closure rate of perimembranous VSD (p‐VSD) and muscular VSD (m‐VSD). Our aim was to seek to the spontaneous closure predictive factors of VSD, and to recommend a follow‐up criteria for assisting pediatrician in selecting the best management scheme for VSD children.

## 2 METHODS AND METHODS

### 2.1 Study population

Between January 2008 and December 2016, 132 children presented with isolated VSD at Shengjing Hospital of China Medical University by echocardiography. Out of 132, 127 patients, 66 males, and 61 females with average age of 7.99 ± 8.52 months, were diagnosed either with p‐VSD or m‐VSD. Five children were excluded from the study during the follow‐up period due to surgical intervention (including 4 p‐VSD and 1 m‐VSD). This study did not include patients with inlet and outlet VSD. Because most patients were recommended for early surgical treatment, rather than follow‐up observation. All participating children underwent echocardiography yearly till the closure of VSD or 6 years old. All parents and guardians were provided written informed consent for the minors to participate in this study. The school ethics committee approved the study.

### 2.2 Echocardiographic parameters

All echocardiographic examinations were performed using Philips iE33 to select S5‐1 (frequency 1.0‐5.0 MHz) or S12‐4 probe (frequency 4.0‐12.0 MHz). Clinical indicators (sex, initial diagnosis age) were collected at the beginning of our study. Since most patients have a first diagnosis age of less than 6 months, they were divided into two groups: less than 6 months and more than 6 months. Then, children patients underwent conventional echocardiography. They were placed in the conventional left lateral position, once asleep or quiet, and parasternal long‐axis, parasternal short‐axis, and apical four‐chamber views were obtained. The main purpose was to scan the ventricular septum completely, and combine with color Doppler echocardiography to determine the type and position of the defects. Defect size measured by 2D echocardiography. If the defect boundary was not clearly displayed, color flow mapping may be used. The diameter of defect was recorded as the maximal VSD diameter in multiple planes. Defect size was divided into three groups^6^: (a) less than 3 mm was classified as small; (b) 3 to 5 mm was classified as medium; (c) more than 5 mm was classified as large. Number of defect were divided into two groups: single defect or multiple defects. Position of m‐VSD was classified as (a) anterior; (b) midmuscular; (c) apical VSD as described in Ramaciotti et al.^9^ According to whether the aneurysms tissue of the ventricular membranous septum (ATVMS) was formed, p‐VSD was divided into two groups. Diagnostic criteria of pulmonary hypertension (PH) were defined as pulmonary artery systolic pressure > 36 mm Hg, or mean pulmonary artery pressure > 25 mm Hg.^10^ We followed the recommended guidelines^11^ to measure left ventricular end‐diastolic dimension (LVDD), aortic root dimension (AOD), and pulmonary artery dimension (PAD). Spontaneous closure was confirmed by color Doppler mapping, and the heart murmur was undetectable.

### 2.3 Establishment of logistic model

The model was generated with spontaneous closure as a dependent variable (spontaneous closure as 1, no closure as 0), defect size (X_1_), ATVMS (X_2_), and LVDD(X_3_) as independent variables.

### 2.4 Statistical analysis

Using SPSS 23.0 software, continuous variables were expressed as mean ± SD (X ± SD), and *t*‐test was used for comparison between groups; dichotomous variables was expressed as percentage, and *χ* ^2^ tests was used for comparison between groups. Statistically significant clinical indicators and echocardiographic parameters were used to identify predictors by binary Logistic Regression analysis. Likelihood ratio test was used to evaluate the fit of the entire model. The predictability of the Logistic Regression model was evaluated by the ROC analyses. The area under the curve >0.5 would predict spontaneous closure, while the area under the curve ≤0.5 would predict open. *P* < 0.05 was considered statistically significant.

## 3 RESULT

Evaluation of 127 children with isolated VSD by echocardiography showed that the frequency of p‐VSD was more common (74 cases, 58%) than m‐VSD (53 cases, 42%). These children patients (66 boys and 61girls), aged 17 days to 48 months (mean 7.99 ± 8.52 months), were followed from 4 months to 74 months (mean 35.20 ± 19.92 months). Spontaneous closure occurred in 76 patients (60%).

### 3.1 P‐ventricular septal defect

**Figure 1:**
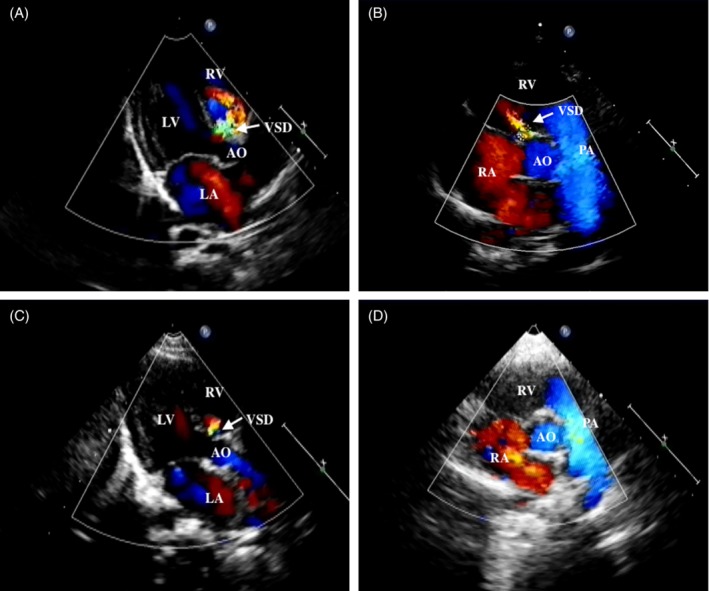
Show a process of ventricular septal defect (VSD) children spontaneously closed by echocardiography. A case of a 32‐days girl baby who underwent echocardiography due to heart murmur. A, Color Doppler echocardiography shows parasternal long‐axis viewed of a perimembranous defect, and the width of defect was 4.0 mm. B, When she was 90 days, color doppler echocardiography showed the defect of the parasternal short‐axis had been reduced, and the width of defect was 3.1 mm. C, When she was 8 months, this defect became smaller to 2.0 mm. D, When she was 14 months, the defect by echocardiography has closed spontaneously, and heart murmur also disappeared. AO, aortic; LA, left atrium; LV, left ventricular; PA, pulmonary artery; RA, right atrium; RV, right ventricular; VSD, ventricular septal defect

Spontaneous closure rate in patients with p‐VSD was 57% (Figure 1). Males had a better closure rate (62%) compared with females (51%); By the number of defect, spontaneous closure rates were shown as follows: (a) single, 39 of 69 (57%); (b) multiple, 3 of 5 (60%). Patients less than 6 months of initial diagnosis age showed a higher rate (69%) than older than 6 months (45%). There was a negative association between defect size and rate of spontaneous closure; small defects had the highest rate (83%). ATVMS was a favorable factor for spontaneous closure. The spontaneous closure rate of p‐VSD with ATVMS was 70%, while that without ATVMS was only 24%. PH was a negative factor for spontaneous closure. Although there were only five patients with PH in this study, none of them were closed. LVDD of spontaneous closure was smaller than no closure. Differences of results between patients with and without spontaneous closure of p‐VSD are summarized in Table 1.

**Table 1:** Differences of results between patients with and without spontaneous closure of perimembranous VSD

Indicator		Spontaneous closure	No closure	*P* value
Total, n (%)		42 (57)	32 (43)	—
Sex, n (%)	Male	23 (62)	14 (38)	0.348
	Female	19 (51)	18 (49)	
Number of defect, n (%)	Single	39 (57)	30 (43)	1.000
	Multiple	3 (60)	2 (40)	
First diagnosis age, n (%)	≤6	25 (69)	11 (31)	0.032*
	>6	17 (45)	21 (55)	
Defect size, n (%)	Small	24 (83)	5 (17)	<0.001*
	Medium	15 (43)	20 (57)	
	Large	3 (30)	7 (70)	
ATVMS, n (%)	Yes	37 (70)	16 (30)	<0.001*
	No	5 (24)	16 (76)	
PH, n (%)	Yes	0 (0)	5 (100)	0.029*
	No	42 (61)	27 (39)	
LVDD (mm)		24.22 ± 4.11	26.89 ± 3.50	0.004*
AOD (mm)		12.38 ± 1.47	13.08 ± 1.75	0.065
PAD (mm)		11.03 ± 1.37	11.72 ± 1.59	0.053

Abbreviations: AOD, aortic root dimension; ATVMS, aneurysms tissue of the ventricular membranous septum; LVDD, left ventricular diastolic dimension; PAD, pulmonary artery dimension; PH, pulmonary hypertension

*P < 0.05.

Binary Logistic Regression analysis identified defect size (*P* = 0.003), ATVMS (*P* = 0.005), and LVDD (*P* = 0.010) as statistically significant indicators that could discriminate spontaneous closure from open.

**Figure 2:**
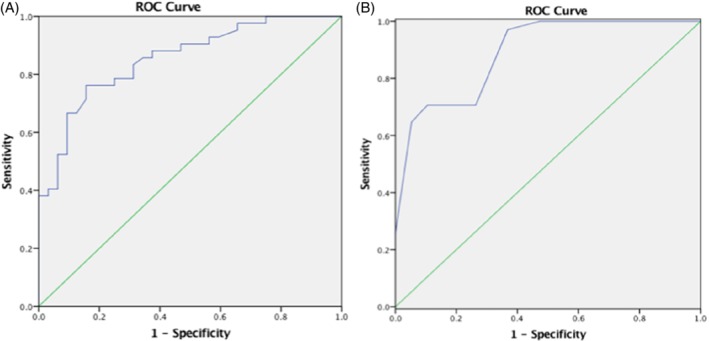
Receiver operating characteristic (ROC) curve of the ability of prognostic factors to predict spontaneous closure of ventricular septal defect (VSD). A, The area under the ROC curve for perimembranous VSD was 0.854 (*P* < 0.001, 95% confidence interval [CI] 0.769, 0.938). B, The area under the ROC curve for muscular VSD was 0.898 (*P* < 0.001, 95% CI 0.814, 0.982). Using a ROC curve, the higher the area under curve the better the accuracy of the test

Constructing a Logistic Regression model using these three indicators as independent variables: Logi t(P) = 7.143 − 1.438X_1_ + 1.887X_2_ − 0.222X_3_. Likelihood ratio test generated χ^2^ = 70.187 (*P* < 0.001) indicating that the model is logical. Using this model, we observed that the correct rate of the prediction results of p‐VSD model was as high as 75.7%. The prediction of spontaneous closure of p‐VSD was assessed by area under the curve. Area under the curve was 0.854 (*P* < 0.001, 95% CI 0.769, 0.938) which reflected effectiveness of the model in predicting the spontaneous closure of p‐VSD (Figure 2).

### 3.2 M‐ventricular septal defect

Spontaneous closure rate of 64% was observed among 53 patients with m‐VSD. As in p‐VSD, males showed a higher rate (66%) compared to females (63%) and 65% of single defects closed spontaneously compared to multiple defects (60%). Spontaneous closure was more frequent in age ≤ 6 months group (83%) vs than in age>6 months (39%) group. The small defects showed the highest frequency (80%) of spontaneous closure. Apical defects closed at a higher rate (67%) than mid‐ventricular (66%) and apical (67%). Similar to p‐VSD, PH was a negative factor for spontaneous closure. Three patients with PH were no closed. LVDD of spontaneous closure was smaller than no closure. Differences of results between patients with and without spontaneous closure of m‐VSD are summarized in Table 2.

**Table 2:** Differences of results between patients with and without spontaneous closure of muscular VSD

Indicator	Spontaneous closure (%)		No closure (%)	*P* value
Total	34 (64)		19 (36)	—
Sex, n (%)	Male	19 (66)	10 (34)	0.552
	Female	15 (63)	9 (37)	
Number of defect, n (%)	Single	31 (65)	17 (35)	1.000
	Multiple	3 (60)	2 (40)	
First diagnosis age,n (%)	≤6	25 (83)	5 (17)	0.001*
	>6	9 (39)	14 (61)	
Defect size, n (%)	Small	32 (80)	8 (20)	<0.001*
	Medium	2 (18)	9 (82)	
	Large	0 (0)	2 (100)	
Position of defect, n (%)	Anterior	3 (50)	3 (50)	0.743
	Mid‐muscular	21 (66)	11 (34)	
	Apical	10 (67)	5 (33)	
PH, n (%)	Yes	0 (0)	3 (100)	0.077
	No	34 (68)	16 (32)	
LVDD (mm)	21.66 ± 5.10		27.40 ± 3.97	<0.001*
AOD (mm)	11.48 ± 2.32		12.89 ± 2.87	0.056
PAD (mm)	10.34 ± 3.22		11.69 ± 1.64	0.094

LVDD, left ventricular diastolic dimension; AOD, aortic root dimension; PAD, pulmonary artery dimension; PH, pulmonary hypertension.

*P < 0.05.

Binary Logistic Regression analysis showed that defect size (*P* = 0.002) and LVDD (*P* = 0.004) statistically discriminated spontaneous closure from open.

A Logistic Regression model was created using the indicators identified by univariate analysis: Logit (P) = 11.216 − 3.150X_1_ − 0.267X_3_. Likelihood ratio test generated χ^2^ = 38.264 (*P* < 0.001), indicating that the model is logical. Application of the Logistic Regression model revealed the frequency of spontaneous closure of m‐VSD was 83%. Area under the curve value was 0.898 (*P* < 0.001, 95% CI 0.814, 0.982), reflecting sensitivity and specificity of the model in predicting the spontaneous closure of m‐VSD (Figure 2).

## 4 DISCUSSION

With the improvement in sensitivity and clarity of 2D combined with color Doppler echocardiography in recent years,^11^ the detection rate of VSD is increased.^3^ However, this has advantages and disadvantages. Echocardiographic follow‐up can improve the understanding of the natural history of VSD, and assess the severity of the disease. Conversely, due to the increase of the detection rate, parents are anxious for VSD children, and pediatricians are also facing great pressure to manage patients with VSD. Our study, which included all the factors in common used and easily acquired might help to predict spontaneous closure of VSD and assist pediatrician in selecting the management scheme for VSD children.

In this study, VSD children were followed up to 6 years old with echocardiography. We thoroughly studied the natural history of p‐VSD and m‐VSD. On the one hand, we found the prevalence of p‐VSD was more common (74 cases, 58%) than m‐VSD (53 cases, 42%). This confirmed the previous observation that the incidence of p‐VSD was higher than m‐VSD.^6^ On the other hand, we demonstrated that the rate of spontaneous closure was 60% in VSD, and m‐VSD are easier closure than p‐VSD, which in similar to the conclusion from Eroglu et al^5^ and Axt‐Fliedner et al.^12^ This difference may be attributed to different closed mechanisms. P‐VSD closure was thought to occur as a result of damage caused by a high‐velocity jet to damage the endothelium at the edge of the VSD or the septal leaflet of the tricuspid valve, both of which can lead to accumulation of platelets and production of fibrous tissue.^13^ On the other hand, the spontaneous closure of m‐VSD was due to muscular encroachment of the septal defect along with superimposed fibrosis or fibrous tissue formation around the margins leading to apposition of the edges of the defect.^4^, ^14^ Another reason may be that m‐VSD size was slightly smaller than p‐VSD. Our study showed the diameter of p‐VSD was 3.43 ± 1.34 mm, and the diameter of m‐VSD was 2.68 ± 1.08 mm.

Although high rate of spontaneous closure was satisfying, there was currently no simple and systematic way to predict spontaneous closure of VSD.^15^ Therefore, it was essential to find out predictors of spontaneous closure of VSD. According to our analysis, defect size, ATVMS, and LVDD were characteristic and representative predictors. Our study showed that defect size was one of the key factors affecting the spontaneous closure of VSD. For the same age of examination, the smaller the defect is, the easier it to close spontaneously. The spontaneous closure rate of small p‐VSD was 83%, and small m‐VSD was 80%. Our results are consistent with these studies,^2^, ^16^ the spontaneous closure probability of small defects was much higher than larger ones. ATVMS formation was also an important factor of the spontaneous closure of p‐VSD. It was reported that the rate of ATVMS formation was 46% to 81%,^2^, ^5^, ^8^ and the large difference depended on the study population. In our study, ATVMS formation was detected in 72% of patients with p‐VSD. And the rate of spontaneous closure was reaching up to 70% in ATVMS. Although the mechanism was still unclear, there were reports that ATVMS formation would limit left‐to‐right shunting and may result in partial or complete closure of VSD.^17^ LVDD was a negative predictor of spontaneous closure, with unfavorable VSD prognosis. Our study showed that LVDD of spontaneous closure was smaller than no closure, which indicates that left‐to‐right shunting was large, and the ventricular with volume overload resulted in left ventricular enlargement. King et al^18^ reported increasing left ventricular size was an indication for surgery closure. Although PH was not correlation with predicting spontaneous closure of VSD in the model, it was negative factor of spontaneous closure. There were only five patients of p‐VSD and three patients of m‐VSD with PH in this study, none of them were closed. Further studies with larger sample size and long follow‐up duration of VSD patients with PH are recommended.

Whether the position of m‐VSD affects the probability potential for spontaneous closure remains debatable. The results of this study indicated that the spontaneous closure of m‐VSD is independent of the position of the defect, which is in line with published reports.^7^, ^19^, ^20^ We observed that the incidence rate of mid‐muscular VSD, which is the most common position for m‐VSD, was 60% with a spontaneous closure rate of 66%. The spontaneous closure rate of apical defects was 67%, higher than the mid‐muscular VSD and the apical VSD. This is contrary to previous reports which showed that apical VSD and anterior VSD persisted longer than mid‐muscular VSD.^7^, ^9^ Possible reasons for this disparity are differences in follow‐up time, diagnostic criteria, and ethnicity.^3^, ^21^

It is well known that spontaneous closure of VSD can occur at any age, but most commonly occurs in childhood.^22^ According to the statistics, the mean age of closure of p‐VSD were 28.88 ± 15.95 months (median 24.50 months), and the mean age of closure of m‐VSD were 28.61 ± 20.14 months (median 26.20 months), indicating that spontaneous closure occurred mainly in the first 3 years. In a study by Yang et al^22^, spontaneous closure of VSD in the first 3 years was accounted for 78.50% of all spontaneous closure events which decreased dramatically after 4 years of age.

Clinical management of isolated VSD is imperative because long‐term persistence of defects may cause the occurrence of complications. Complications such as infective endocarditis, LV volume overload,^23^, ^24^ double‐chambered right ventricle, and arrhythmia may develop over time.^25^ Appropriate follow‐up of patients with VSD may assist the pediatrician in understanding of patients condition and disease change. Convenient and efficient echocardiography is used to measure the predictors of the spontaneous closure of VSD. It is helpful for pediatrician to assess the spontaneous closure of VSD and reduce parental anxiety. This can also help pediatrician to detect the complications as soon as possible, and treat children with bad conditions timely to improve the prognosis of patients.^2^, ^26^ Our model had good discriminatory ability. The areas under the curve of predicting p‐VSD and m‐VSD model was 0.854 and 0.898, respectively. Based on the findings of this study, we recommend the follow‐up criteria for patient with isolated p‐VSD and m‐VSD:

1. Patients with small VSD: These patients have the high possibility of spontaneous closure. If patients have ATVMS formation and no LVDD increased, the spontaneous closure may be more frequent. In patients with asymptomatic and no complications, surgery may be not recommended. It is reasonable to follow‐up regularly for every 1 to 2 years.
2. Patients with medium VSD: These patients have the intermediate possibility of spontaneous closure. Similarly, ATVMS formation and no LVDD increased are good predictors. Once a year review is reasonable. Data should be obtained include change in defect size, presence or absence of complications, PH, and LV volume overload. Once presence, appropriate treatment strategy may be beneficial. And parents may be mentally as well as economically prepared.
3. Patients with large VSD: These patients have low possibility of spontaneous closure. There is usually LV volume overload, PH, and even congestive heart failure and Eisenmenger syndrome in infancy or childhood. It is probably recommended that patients can be seen at a CHD center of at least yearly. Patients with complications and poor lesion should be considered for surgery.

## 5 LIMITATION

There were many limitations in this study. (a) The number of patients was not big enough, and the follow‐up time was not long enough. The validation of the model requires more cases and longer follow‐up. (b) This study did not combine other clinical examinations and laboratory indicators. Therefore, our information can be used to predict the possibility of spontaneous closure and decide treatment options, but not to provide an accurate diagnosis.

## 6 CONCLUTION

This study described the natural history and spontaneous closure rate of children with p‐VSD and m‐VSD. We inferred that defect size, ATVMS and LVDD were characteristic and representative predictors for spontaneous closure of VSD. A follow‐up criteria was recommended by summarizing this study, which may assist pediatricians in selecting the appropriate treatment strategy to manage VSD children.

## CONFLICT OF INTEREST

The authors declare no potential conflict of interests.

## References

[1] Cho YS , Park SE , Hong SK , Jeong NY , Choi EY . The natural history of fetal diagnosed isolated ventricular septal defect.Prenat Diagn. 2017;37(9):889‐893. 28639332 10.1002/pd.5100

[2] Ricci Z , Sun J , Sun K , Chen S , Yao L , Zhang Y . A new scoring system for spontaneous closure prediction of Perimembranous ventricular septal defects in children.PLoS One. 2014;9(12):e113822. 10.1371/journal.pone.0113822 PMC4257539 25479616

[3] Penny DJ , Vick GW III . Ventricular septal defect.Lancet. 2011;377(9771):1103‐1112. 21349577 10.1016/S0140-6736(10)61339-6

[4] Li X , Song GX , Wu LJ , et al. Prediction of spontaneous closure of isolated ventricular septal defects in utero and postnatal life.BMC Pediatr. 2016;16(1):207. 27931195 10.1186/s12887-016-0735-2 PMC5146819

[5] Eroglu AG , Atik SU , Sengenc E , Cig G , Saltik IL , Oztunc F . Evaluation of ventricular septal defect with special reference to the spontaneous closure rate, subaortic ridge, and aortic valve prolapse II.Pediatr Cardiol. 2017;38(5):915‐921. 28401252 10.1007/s00246-017-1597-6

[6] Cresti A , Giordano R , Koestenberger M , et al. Incidence and natural history of neonatal isolated ventricular septal defects: do we know everything? A 6‐year single‐center Italian experience follow‐up.Congenit Heart Dis. 2018;13(1):105‐112. 28857497 10.1111/chd.12528

[7] Miyake T , Shinohara T , Inoue T , Marutani S , Takemura T . Spontaneous closure of muscular trabecular ventricular septal defect: comparison of defect positions.Acta Paediatr. 2011;100(10):e158‐e162. 21517965 10.1111/j.1651-2227.2011.02333.x

[8] Miyake T , Shinohara T , Fukuda T , Ikeoka M , Takemura T . Spontaneous closure of perimembranous ventricular septal defect after school age.Pediatr Int. 2008;50(5):632‐635. 19261109 10.1111/j.1442-200X.2008.02642.x

[9] Ramaciotti C , Vetter JM , Bornemeier RA , Chin AJ . Prevalence, relation to spontaneous closure, and association of muscular ventricular septal defects with other cardiac defects.American Journal of Cardiology. 1995;75(1):61‐65. 7801866 10.1016/s0002-9149(99)80529-3

[10] Sciomer S , Magrì D , Badagliacca R . Non‐invasive assessment of pulmonary hypertension: Doppler–echocardiography.Pulm Pharmacol Ther. 2007;20(2):135‐140. 16753319 10.1016/j.pupt.2006.03.008

[11] Lang RM , Badano LP , Mor‐Avi V , et al. Recommendations for cardiac chamber quantification by echocardiography in adults: an update from the American Society of Echocardiography and the European Association of Cardiovascular Imaging.Eur Heart J Cardiovasc Imaging. 2015;16(3):233‐270. 25712077 10.1093/ehjci/jev014

[12] Axt‐Fliedner R , Schwarze A , Smrcek J , Germer U , Krapp M , Gembruch U . Isolated ventricular septal defects detected by color Doppler imaging: evolution during fetal and first year of postnatal life.Ultrasound Obstet Gynecol. 2006;27(3):266‐273. 16485323 10.1002/uog.2716

[13] Nir A , Driscoll DJ , Edwards WD . Intrauterine closure of membranous ventricular septal defects: mechanism of closure in two autopsy specimens.Pediatr Cardiol. 1994;15(1):33‐37. 8115270 10.1007/BF00797004

[14] Nir A , Weintraub Z , Oliven A , Kelener J , Lurie M . Anatomic evidence of spontaneous intrauterine closure of a ventricular septal defect.Pediatr Cardiol. 1990;11(4):208‐210. 2274448 10.1007/BF02238368

[15] Stonawski V , Vollmer L , Kohler‐Jonas N , et al. Long‐term associations of an early corrected ventricular septal defect and stress Systems of Child and Mother at primary school age.Front Pediatr. 2017;5:293. 29379779 10.3389/fped.2017.00293 PMC5775274

[16] Gersony WM . Natural history and decision‐making in patients with ventricular septal defect.Progress in Pediatric Cardiology. 2001;14(2):125‐132.

[17] Warnes CA , Williams RG , Bashore TM , et al. ACC/AHA 2008 guidelines for the Management of Adults with Congenital Heart Disease.Circulation. 2008;118(23):e714‐833. 10.1161/CIRCULATIONAHA.108.190690 18997169

[18] King ME , de Moor M . Ventricular septal defect.Curr Treat Options Cardiovasc Med. 1999;1(4):311‐322. 11096497 10.1007/s11936-999-0026-4

[19] Chang JK , Jien WY , Chen HL , Hsieh KS . Color Doppler echocardiographic study on the incidence and natural history of early‐infancy muscular ventricular septal defect.Pediatr Neonatol. 2011;52(5):256‐260. 22036220 10.1016/j.pedneo.2011.06.003

[20] Erol O , Sevket O , Keskin S , Yazicioglu HF , Gul A . Natural history of prenatal isolated muscular ventricular septal defects.J Turk Ger Gynecol Assoc. 2014;15(2):96‐99. 24976775 10.5152/jtgga.2014.0012 PMC4072558

[21] Van dHF , Timmers T , Hess J . Morphological, haemodynamic, and clinical variables as predictors for management of isolated ventricular septal defect.Br Heart J. 1995;73(1):49. 7888261 10.1136/hrt.73.1.49 PMC483755

[22] Xu Y , Yang S . Factors influencing the spontaneous closure of ventricular septal defect in infants.Int J Clin Exp Pathol. 2014;64(16):C192‐C193. PMC4503144 26191273

[23] Habib G , Lancellotti P , Antunes MJ , et al. 2015 ESC guidelines for the management of infective endocarditis: the task force for the Management of Infective Endocarditis of the European Society of Cardiology (ESC) Endorsed by: European Association for Cardio‐Thoracic Surgery (EACTS), the European Association of Nuclear Medicine (EANM).Eur Heart J. 2015;36(44):3075‐3128. 26320109 10.1093/eurheartj/ehv319

[24] Moscarelli M , Attaran S , Thomas C , Anderson JR . Contained rupture of the sinus of valsalva associated with infective endocarditis and untreated congenital ventricular septal defect.World J Pediatr Congenit Heart Surg. 2013;4(3):312‐314. 24327505 10.1177/2150135112474025

[25] Baumgartner H , Bonhoeffer P , De Groot NMS , et al. ESC guidelines for the management of grown‐up congenital heart disease (new version 2010): the task force on the Management of Grown‐up Congenital Heart Disease of the European Society of Cardiology (ESC).Eur Heart J. 2010;31(23):2915‐2957. 20801927 10.1093/eurheartj/ehq249

[26] Karonis T , Scognamiglio G , Babu‐Narayan SV , et al. Clinical course and potential complications of small ventricular septal defects in adulthood: late development of left ventricular dysfunction justifies lifelong care.Int J Cardiol. 2016;208:102‐106. 26844920 10.1016/j.ijcard.2016.01.208

